# Distribution of Serotypes, Vaccine Coverage, and Antimicrobial Susceptibility Pattern of Streptococcus Pneumoniae in Children Living in SAARC Countries: A Systematic Review

**DOI:** 10.1371/journal.pone.0108617

**Published:** 2014-09-30

**Authors:** Nishant Jaiswal, Meenu Singh, Rashmi Ranjan Das, Ishita Jindal, Amit Agarwal, Kiran Kumar Thumburu, Ajay Kumar, Anil Chauhan

**Affiliations:** 1 ICMR Advanced Centre for evidence based Child Health, Department of Pediatrics, Post-Graduate Institute of Medical Education & Research, Chandigarh, India; 2 Department of Pediatrics, Post-Graduate Institute of Medical Education & Research, Chandigarh, India; 3 Department of Pediatrics, All India Institute of Medical Sciences, Bhubaneswar, India; 4 Department of Ophthalmology, Wayne State University, Detroit, Michigan, United States of America; Instituto Butantan, Brazil

## Abstract

**Introduction:**

Each SAARC nation falls in the zone of high incidence of pneumococcal disease but there is a paucity of literature estimating the burden of pneumococcal disease in this region.

**Objective:**

To identify the prevalent serotypes causing invasive pneumococcal disease in children of SAARC countries, to determine the coverage of these serotypes by the available vaccines, and to determine the antibiotic resistance pattern of *Streptococcus pneumoniae*.

**Methods:**

We searched major electronic databases using a comprehensive search strategy, and additionally searched the bibliography of the included studies and retrieved articles till July 2014. Both community and hospital based observational studies which included children aged ≤12 years as/or part of the studied population in SAARC countries were included.

**Results:**

A total of 17 studies were included in the final analysis. The period of surveillance varied from 12–96 months (median, 24 months). The most common serotypes country-wise were as follows: serotype 1 in Nepal; serotype 14 in Bangladesh and India; serotype 19F in Sri Lanka and Pakistan. PCV-10 was found to be suitable for countries like India, Nepal, Bangladesh, and Sri Lanka, whereas PCV-13 may be more suitable for Pakistan. An increasing trend of non-susceptibility to antibiotics was noted for co-trimoxazole, erythromycin and chloramphenicol, whereas an increasing trend of susceptibility was noted for penicillin.

**Conclusion:**

Due to paucity of recent data in majority of the SAARC countries, urgent large size prospective studies are needed to formulate recommendations for specific pneumococcal vaccine introduction and usage of antimicrobial agents in these regions.

## Introduction

*Streptococcus pneumoniae* or *Pneumococcus* claims 1 million child deaths every year worldwide [1]. Approximately 90% of these deaths occur in developing countries. A recent systematic analysis reported that, of 7.6 million deaths in children younger than 5 years in 2010, pneumonia accounted for 1.071 million (14·1%) deaths [2]. For every 1 child that dies of pneumonia in a developed country, more than 2000 children die of pneumonia in developing countries [3]. Besides pneumonia, *S. pneumoniae* causes a wide spectrum of diseases including pharyngitis, acute otitis media, joint effusions, meningitis, bacteremia and/or septicemia.

The SAARC (The South Asian Association for Regional Cooperation) includes 8 countries: India, Pakistan, Bangladesh, Sri Lanka, Nepal, Bhutan, Maldives, and Afghanistan. The under-five mortality rates (per 1000 live-births) are high in these regions (ranging from 10 for Sri Lanka to 99 for Afghanistan) compared to the western countries (UK = 5, and USA = 7) as per the 2012 WHO data. The share of under-five deaths due to pneumonia in these regions is as follows: 20.4% in Afghanistan; 15% in India, 14.6% in Pakistan, 13.6% each in Nepal and Bhutan; 11% in Bangladesh, 8.8% in Maldives, and 5.7% in Sri Lanka [2], [4]. The SAARC nations also fall in the zone with high incidence of pneumococcal disease [1], but there is a dearth of studies reporting prevalent serotypes in these regions.

Different pneumococcal serotypes show different antibiotic sensitivity, and most of them are now resistant to the commonly prescribed antibiotics. Both, overuse of antibiotics and their over-the-counter availability have contributed to the increasing antibiotic resistance. In order to combat the increasing incidence of resistance as well as increasing disease prevalence, pneumococcal vaccines were made available as preventive tools. Since the availability of the first 23-valent-polysaccharide pneumococcal vaccine (PPV-23) in 1977, many new conjugate vaccines (PCV-7, PCV-10, PCV-13) have been introduced and tested, but no single vaccine covers all 90 known pneumococcal serotypes [5]. These vaccines constitute those strains that cause 80% of the invasive pneumococcal disease (IPD) and are resistant to antibiotics [5].

WHO-GAVI (World Health Organization & Global Alliance for Vaccines and Immunisation) alliance has approved 3 conjugate vaccines PCV-7; PCV-10, and PCV-13 for use in children. These vary in the serotypes contained and the proteins used for conjugation. Vaccine serotypes are categorized based on the following vaccine preparations: 7 valent — 4, 6B, 9V, 14, 18C, 19F, and 23F; 10 valent — 1, 4, 5, 6B, 7F, 9V, 14, 18C, 19F, and 23F; and 13 valent — 1, 3, 4, 5, 6A, 6B, 7F, 9V, 14, 18C, 19A, 19F, and 23F. The introduction of these vaccines in The United States (US) and Western Europe has decreased the incidence of vaccine strain associated invasive pneumococcal disease (IPD) significantly. The GAVI alliance has also identified 75 low & middle-income countries that include the SAARC countries, to aid in vaccine introduction. The dilemma faced by SAARC countries is “which pneumococcal vaccine to introduce?” Pakistan is the only country from SAARC, where PCV-10 has been introduced with the help of GAVI alliance [6]. Both, estimates of pneumococcal disease burden along with antibiotic resistance pattern as well as knowledge about the prevalent serotypes are needed, to utilize the resources for child survival such as available vaccines and antibiotic therapy in other SAARC countries.

## Methods

### Types of studies

Observational studies (prospective, retrospective) reporting data on different *S. pneumoniae* serotypes obtained from normally sterile sites (e.g. blood, cerebrospinal fluid, pleural fluid) after at least 12 months of surveillance to avoid seasonal variation in reporting of the serotypes were included. The studies, commenting only on antibiotic resistance, without isolating the causative organism, were excluded. We also excluded case reports, editorials, vaccine studies, literature reviews and the studies in which nasopharyngeal aspirates, throat swabs or oro-pharyngeal swabs were the only samples to determine the causative organism.

### Types of participants

Participants were children of both sexes and ≤12 years of age (excluding the neonates or young infants <2 months) as studied population in the SAARC countries.

### Outcome measures

We intended to analyze the serotype distribution and pattern of antimicrobial resistance among *S. pneumoniae* isolates causing IPD in SAARC countries so as to provide guidance regarding immunization. So, the following outcomes of interest were measured.

*Primary outcome*

1. Prevalence of different invasive pneumococcal serotypes

*Secondary outcome*

1. Antibiotic resistance pattern of *S. pneumoniae*
2. Vaccine serotype coverage rate with currently available pneumococcal vaccines

### Search methodology

We performed a systematic search of the published literature and also tried to acquire information about the unpublished literature from various investigators of the region. The searches were conducted from year 1970 to July 2014, and we identified articles with information on IPD among children ≤12 years of age. We searched following databases: Medline via Ovid, Pubmed, Embase and The Cochrane library (details of search strategy has been provided in **Appendix S1**). Non English articles were not included. Searches were carried out by two authors (NJ, RRD). After the search, each author was advised to screen the titles and abstracts for eligibility, and to retrieve full text articles. In case of any disagreement, a consensus was reached after discussion with the third author (MS). If the required data was not available we contacted the authors and tried to resolve discrepancies.

### Data extraction

Authors abstracted data separately from the included studies in a predesigned proforma that included author, date of publication, country of study, study setting, population studied, type of study, source of isolates, serotypes isolated, time period of study, antibiotic susceptibility testing method, and antibiotic non-susceptibility rates. Susceptibility data were extracted for the following antibiotics where available: penicillin/ampicillin/amoxicillin, erythromycin, co-trimoxazole, chloramphenicol, ceftriaxone/cefotaxime, and ciprofloxacin. Non-susceptibility comprised of both intermediate and high level non-susceptibility. The proforma was pilot tested before extracting any study data following which data was abstracted separately for hospital-based and population-based studies. To resolve the discrepancies regarding the abstracted data, a consensus was made after discussion with the arbitor (MS).

### Data analysis

After data extraction, all the relevant data was entered into Microsoft Excel. The percentages (%) of each serotype from similar studies of a country were combined together to find the ‘percentage incidence’ of that serotype for that country. We also combined the result from all the SAARC countries to find the most common serotype distribution and the most suitable of the three pneumococcal conjugate vaccines. Antimicrobial susceptibility pattern was studied overall as well as in subgroups with respect to the country, age group, and time period of study, by taking an average estimate of the recent data from similar studies of a particular or all the SAARC countries.

## Results

**Figure 1:**
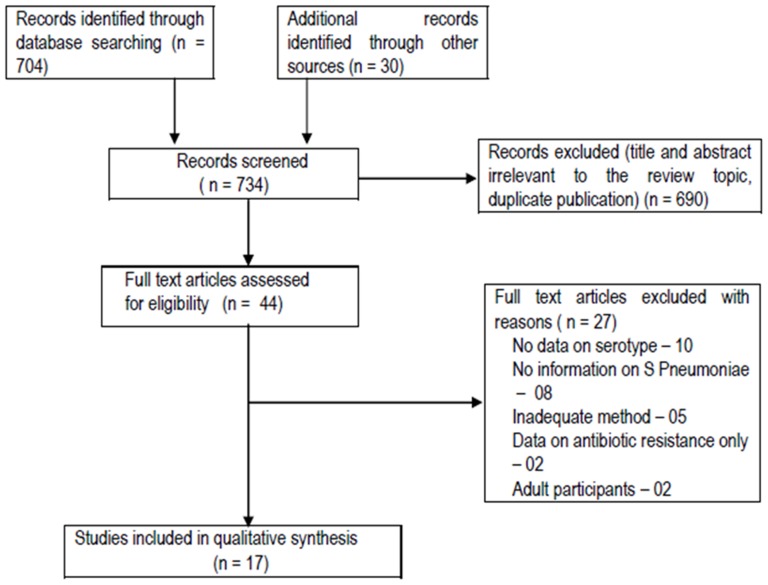
Flow of study in the review.

A total of 734 articles were retrieved, of which 44 articles were found eligible (**Figure 1**). After going through the full text, we were able to include 17 studies (**Table 1**) [7]–[23]. The reasons for exclusion of studies are mentioned in **Table 2**. The data from two Indian studies [10], [11] were not included in the analysis of serotype data as they reported S. pneumoniae serogroups instead of serotypes. But the data from these two studies were included in other analyses. The studies included children ≤12 years of age, and spanned over a period of 22 years (1991–2013) with the surveillance period varying from 12 to 96 months (median, 24 months). Of the 17 included studies, 6 were from Bangladesh, 4 from Nepal, 4 from India, and 2 from Pakistan, and 1 from Sri Lanka. Thirteen were hospital based [7]–[11], [15]–[20], [22], [23], two (conducted in Bangladesh) were population based [13], [14], and two ware combined hospital and population based prospective studies [12], [21]. All the studies used culture and/or antigen detection method for isolation of the organism either from blood, CSF or both, and one study also used pleural fluid. We could not find any eligible studies from three other SAARC countries (Bhutan, Afghanistan and Maldives) to be included in the analysis.

**Table 1:** Characteristics of included studies.

Study ID	Settings	Country	Study period (year), duration (months)	Total children studied	No of S. Pneumoniae positive cases	Diagnosis method	Serotypes isolated	Sample used
Mastro et al, 1991 [7]	Hospital based prospective study	Pakistan	1986–1989, 12 months	NA	87	Culture	1, 5, 6a, 6B, 9V, 15C, 16, 19A, 19F, 31	Blood
Saha et al, 1997 [8]	Hospital based prospective study	Bangladesh	1992–1995, 36 months	NA (<5 yr age)	165	Culture and antigen testing	1, 4, 7F, 12F, 14, 15B, 23F,18, 22A, 25, 16F, 6A, non-typeable	Blood and CSF
Saha et al, 1999 [9]	Hospital based prospective study	Bangladesh	1993–1997, 48 months	NA (<5 yr age)	362	Culture and antigen testing	1, 2, 5, 6, 7F, 9V, 12, 14, 15, 18, 19, 20, 33, 45	Blood, CSF, ear, eye and pus swabs
IBIS Phase-I, 1999 [10]	Hospital based prospective study	India	1993–1997, 48 months	4348 (<12 yr age)	156 (<12 yr age); 103 (<5 yr age)	Culture and antigen testing	1, 6, 15, 7, 19, 5, 18,4, 23, 9, 10, 11, 12, 14, 29, 45, 24, 33, 2	Blood and CSF
IBIS Phase-II, 2002 [11]	Hospital based prospective study	India	2000–2001, 21 months	1764 (<12 yr age)	183	Culture and antigen testing	1, 6, 7, 19, 5, 4, 14, 18, 3	Blood and CSF
Saha et al, 2003 [12]	Hospital and Population based prospective study	Bangladesh	1999–2000, months	2839 (<5 yr age)	1301 (invasive = 91)	Culture and antigen testing	2, 1, 14, 45, 5, 7, 12, 18, 19, 33, 6, 46	CSF
Brooks et al, 2007[13]	Population based prospective study	Bangladesh	2004–2006, 24 months	6167 (<5 yr age)	34	Culture	1, 10F, 12A, 12F, 14, 18A, 18C, 18F, 19A, 19F, 2, 23F, 25, 38, 4, 45, 5, 6B, 9V	Blood
Arifeen et al, 2009 [14]	Population based prospective study	Bangladesh	2004–2007, 36 months	22,378 (<5 yr age) (hospitalized = 2596)	26	Culture	1, 5, 10F, 12A, 14, 18B, 18C, 19A, 35B, 38, 45	Blood and CSF
Shah et al, 2009 [15]	Hospital based prospective study	Nepal	2004–2007, 29 months	2528 (<5 yr age)	51	Culture and antigen testing	1, 5, 2, 7F, 12A, 14, 16, 18F, 19, 19B, 19F, 23F, 32, 39, 6B, non-typeable	Blood and CSF
Willams et al, 2009 [16]	Hospital based prospective study	Nepal	2005–2006, 21 months	885 (<5 yr age)	17	Culture and antigen testing	1, 2, 5, 8, 12A, 16, 18C, 19A, 33, 35,42, non-typeable	Blood and CSF
Batuwanthudawe et al, 2009 [17]	Hospital based prospective study	Srilanka	2005–2007, 26 months	3642 (<5 yr age)	23	Culture and antigen testing	19F, 14, 23F, 6B, 3, 16, 20, 29, 15B, 35, 42, non-typeable	Blood and CSF
Saha et al, 2009 [18]	Hospital based prospective study	Bangladesh	2004–2007, 36 months	17,969 (<5 yr age)	139	Culture and antigen testing	2, 1, 14, 45, 5, 7F, 12A, 18C, 19A, 23F, 6A, 6B, 19F, 18F, 18A, 23A, 24, 29, 4, 20, 33F, 12F, 15B, 15A, 18A, 21, 23B, 3, 33B, 35F, 35A, 48, 8, 9V, 16F, 10F	Blood and CSF
Rijal B et al, 2010 [19]	Hospital based prospective study	Nepal	2004–2008, 50 months	3774	60	Culture and antigen testing	1, 39, 5, 16, 32, 2, 14, 18F, 23F, 7F, 19B, 12A, 19F, 6B, 25F	Blood, CSF and pleural fluid
Kelly et al, 2011 [20]	Hospital based prospective study	Nepal	2005–2006, 21 months	2039 (≤12 yr age)	22	Culture and antigen testing	1, 12A, 5, 9A, 9V, 2, 8, 15A, 16, 18C, 19A, 19C, 23B, 25F, 33, non-typeable	Blood and CSF
Manoharan et al, 2013 [21]	Hospital/community based prospective study	India	2-11-2013,? 24 months	3572 (<5 yr age)	225	Culture and antigen testing	14, 5, 1, 19F, 6B, 2, 7B, 7C, 8, 9A, 10A, 10F, 12F, 15A, 15B, 15C, 15F, 16F, 17F, 18A, 18B, 18F, 19B, 21, 23A, 24A, 24F, 25A, 27, 28F, 31, 33A, 33B, 33F, 34, 35A, 35B, 35F, 36, 38, 45, 6C	Blood, CSF and pleural fluid
Shakoor et al, 2013 [22]	Hospital/community based prospective study	Pakistan	2005–2013,? 96 months	72 (<5 yr age)	111	Culture and antigen testing, PCR	18A, 18B, 18C, 18F, 14, 19F, 23B, 12F, 12A, 44, 46, 5, 9V, 9A, 1, 15B, 15C, 10A, 6A, 6B, 6C, 4, 23F, 19A, 17, 8, 24A, 24B, 24F, 33F, 33A, 37, 35B, 11A, 11D, 22A, 22F, 5, 23A, 23F, 3, 10F, 10C, 33C, 35B, 7A, 7F, 13, 38, 25F, 25A; no-typeable	Blood, CSF and pleural fluid
Kumar et al, 2013 [22]	Hospital/community based prospective study	India	2-11-2013,? 24 months	3572 (<5 yr age)	225	Culture and antigen testing	1,; 3; 4; 5; 6; 9; 10; 14; 15; 18; 19	Blood, CSF and pleural fluid

NA: Not available, CSF: Cerebro-spinal fluid, Numbers in brackets includes references

**Table 2:** Excluded studies.

Study name	Reasons for exclusion
Patwari et al, 1988	No available data on causative organism
Mastro et al, 1993	Study period is <1 year; Nasopharyngeal aspirates only
Awasthi et al, 1997	No data on *S. pneumoniae*
Saha et al, 1999	Mentions about antibiotic resistance only
Jebaraj et al, 1999	Nasopharyngeal colonization study
Bansal et al, 2002	Study on adults
Acharya et al, 2003	Does not report for *S. pneumoniae*
Mehta et al, 2003	Tells about Antibiotic resistance only does not give the details of *S. pneumoniae* and other causative organism
Bansal et al, 2006	Not reported *S. pneumoniae* so cannot be included
Bharti et al, 2006	No information on *S. pneumoniae*
Hussain et al, 2006	Cost of treatment study
Nizami et al, 2006	Oropharyngeal aspirate only
SPEAR study, 2008	Study does tell only about India but has included other regions which are not a part of SAARC. Randomized Control Trial
Agarwal et al, 2009	Short report; No data for *S. pneumoniae*
Mathisen et al 2010	Study on viruses
Zaidi et al, 2009	No data on serotyping
Naheed et al, 2009	No serotype data
Vishwanath et al, 2007	No serotype data
Kabra et al, 2003	No serotype data
Sahai et al, 2001	No serotype data
Shameem et al, 2008	No serotype data
Patwari et al, 1996	No serotype data
Bahl et al, 1995	No serotype data
Deivnayagam, 1992	No serotype data
John et al, 1991	Study on viruses
Thomas et al, 2013	Included patients>18 yrs age
Saha et al, 2009	No data on serotype
Owais et al, 2010	No data on serotype

### Overall distribution of serotypes

The combined result from all the SAARC countries showed the most common serotypes to be as follows: serotype 1 in Nepal; serotype 14 in Bangladesh and India; serotype 19F in Sri Lanka and Pakistan.

### Hospital based studies

**Figure 2:**
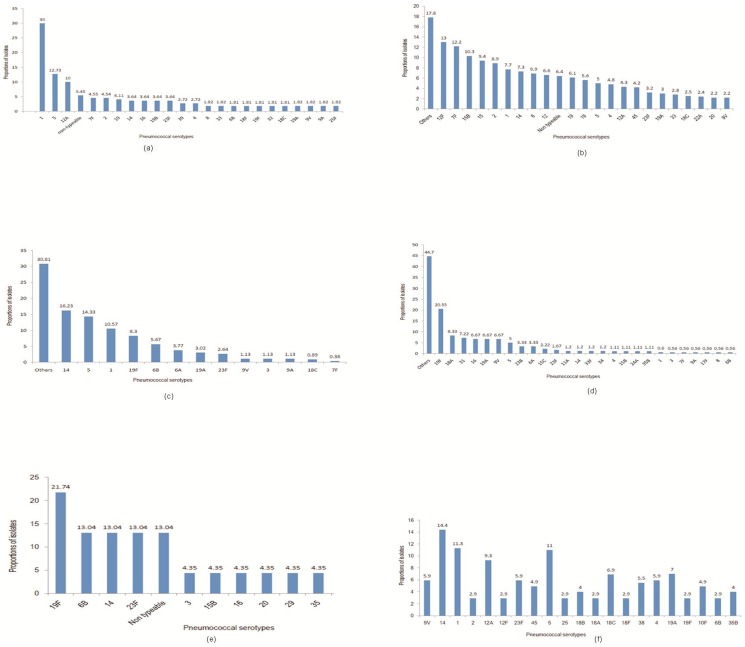
Combined serotype data from (a) Nepal; (b)Bangladesh (hospital based); (c): India; (d): Pakistan; (e): Sri Lanka (f): Bangladesh (population based).

The combined data of the 4 studies from Nepal showed serotype 1 was most common followed by 5, and 12A (**Figure 2a**). Other vaccine serotypes 4, 6A, 6B, 7F, 9V, 14, 18C, 19A, 19F, and 23F were less common. Vaccine serotype 3 was not reported.

The combined data of the 4 Bangladesh studies showed that “other serotypes” were most common. Among the identified ones, 12F, 7F, 15B, 15, 2, 1, and 14 were more common (**Figure 2b**). Other vaccine serotypes 4, 5, 6A, 6B, 9V, 18C, 19A, 19F, and 23F were less common. Vaccine serotype 3 was not reported.

The data from 2 Indian studies showed that “other serotypes” were most common. Among the identified ones, 14 was the most common followed by 5, 1, 19F, and 6B (**Figure 2c**). Other vaccine serotypes 6A, 19A, 23F, and 9V were less common. Only the vaccine serotype 4 was not reported.

The data from 2 Pakistan studies showed that “other serotypes” were most common. Among the identified ones, 19F was the most common followed by 18A, 31, 16, 19A, 9V, and 5 (**Figure 2d**). Other vaccine serotypes 1, 5, 6B, 14, and 23F were less common. Vaccine serotype 2 was not reported.

The data from 1 Sri Lankan study showed serotype 19F to be the most common followed by 6B, 14, 23F, and non-typeable (**Figure 2e**). Other vaccine serotypes 3, 15B, 16, 20, 29, and 35 were less common. Serotypes 1, 2, 4, 5, 6A, 7F, 9V, 18C, and 19A were not reported.

### Population based studies

There were two population based prospective studies from Bangladesh [13], [14]. The combined data showed serotype 14 to be the most common, followed by 1, 5, 12A, 19A, and 18C (**Figure 2f**). Other vaccine serotypes 4, 6B, 23F, 9V, and 19F were less common. Vaccine serotypes 3, 6A, and 7F were not reported.

### Antimicrobial susceptibility

**Figure 3:**
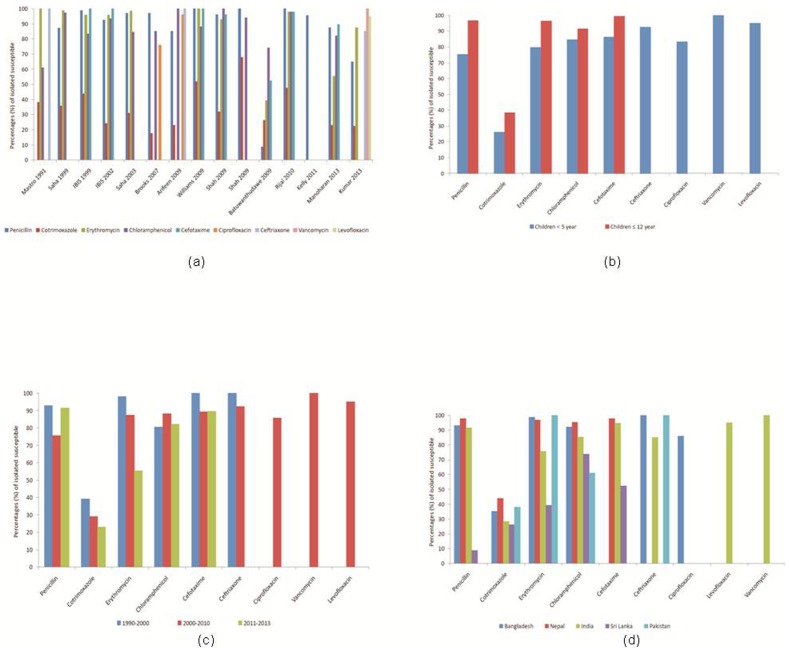
Antibiotic susceptibility pattern of *S Pneumoniae* (a) isolated from all the included studies; (B) Age wise; (C) Year wise; (d) Country wise.

Fifteen studies reported antimicrobial susceptibility pattern of various pneumococcal serotypes. Antimicrobial susceptibility rate of 100% was noted to vancomycin, 95% to levofloxacin, 85–100% to ceftriaxone; 9–98% to penicillin, 40–100% to erythromycin, 53–98% to cefotaxime; 61–95% to chloramphenicol, and 86% to ciprofloxacin. Resistance to co-trimoxazole varied from 56–74% in different studies (**Figure 3a**). Subgroup analysis was done according to the age and the period of study. The mean susceptibility rate was slightly less in children <5 years compared to ≤12 years (**Figure 3b**). We compared the mean susceptibility rate for three different time periods (1990–2000, 2001–2010, and 2011–2013). A decreasing trend (increased non-susceptibility) was noted for co-trimoxazole, erythromycin, and ceftriaxone; cefotaxime showed no change, whereas an increasing trend was noted for penicillin, and chloramphenicol (**Figure 3c**).

### Vaccine serotype coverage

**Figure 4:**
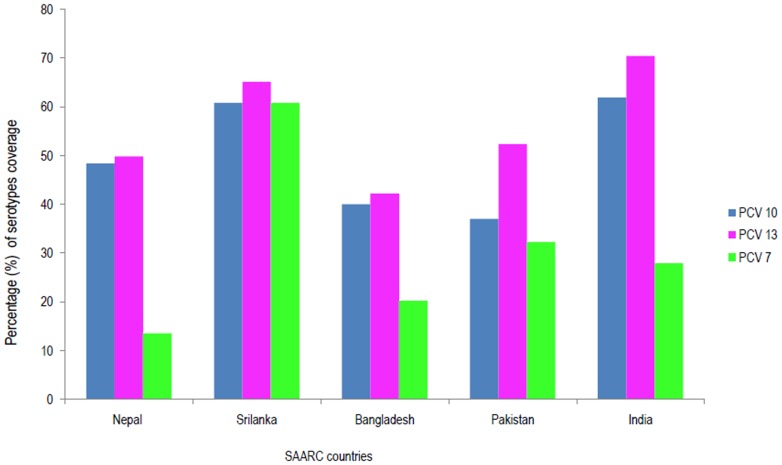
Pneumococcal vaccine coverage as per the serotype isolated (country-wise data).

Based on the prevalent serotypes, we tried to estimate the percentage coverage of various pneumococcal vaccine serotypes in the SAARC countries (**Figure 4**). PCV 13 (13-valent) was found to be more suitable for most of the SAARC countries as it covered three extra serotypes (3, 6A, and 19A) causing IPD compared to PCV 10 (10-valent) and six extra serotypes causing IPD compared to PCV 7 (7-valent). But if we take into account all the parameters including the prevalence of three vaccine serotypes covered by PCV-13 along with the cost as well as cross-protection against related serotypes, then the true difference between PCV-13 and PCV-10 will be minimal. The same has been discussed below in detail.

## Discussion

### Summary of evidence

In the present study, we found the most common serotypes (country-wise) as follows: serotype 1 in Nepal; serotype 14 in Bangladesh and India; serotype 19F in Sri Lanka and Pakistan. Our results show that the cumulative burden of common non-vaccine serotypes (12A, 7, non-typeable, 12F, 15, 31, 2, 19B, 12, 9, 38, 15B, 16, 10F, 45, 35, 29, 18, and 18B) is equal or more than the cumulative burden of the vaccine serotype causing IPD in the SAARC countries. Our results are consistent with the previous systematic review in which the authors found 7 serotypes (1, 5, 6A, 6B, 14, 19F, 23F) to be the most common globally as per the data till July 2007 [24].

It is always a better idea to report the burden of pneumococcal disease separately for each country, so that a particular vaccine can be employed to target the common serotypes prevalent in that region. As shown in the result section, the hospital based data varied from country to country. From all these data, it is assumed that only 13-valent vaccine can cover most of the serotypes depending upon the country setting in SAARC region. Actually the difference between PCV-10 and PCV-13 would not be much if we consider the following points. *First*, PCV-13 covers three extra vaccine serotypes (3, 6A, and 19A). Serotype 3 has not been reported from two countries (Nepal, and Bangladesh), whereas the prevalence is less common in Pakistan (around 1%), India (1.8%) and Sri Lanka (4.3%). *Second*, there is substantial evidence for cross-reactivity among serotypes 6A and 6B. *Third*, emerging evidence also suggests cross-reactivity among serotypes 19A and 19F [25], [26]. The prevalence of serotype 19A is less common in three of the SAARC countries (Nepal = 1.8%, India = 4.8%, Bangladesh = 5%). Pakistan reports a prevalence of 12.5%, whereas Sri Lanka does not report it. *Fourth*, the cost of each dose of PCV-10 is almost half of that of PCV-10. But there is no published literature regarding the cost-effective analysis of PCVs in the SAARC region. Studies from other developing countries and developed countries conclude differently with some studies finding either PCV-10 [27] or PCV-13 [28], [29] or both [30]–[33] to be cost-effective. By taking into account of all these points, it seems that PCV-10 would be suitable for countries like India, Nepal, Bangladesh, Sri Lanka, and Pakistan.

In a previous systematic review [24], combined data from Asian countries showed that the PCV-10 coverage is 70% (95% CI, 64%–75%), and the PCV-13 coverage is 75% (67%–79%). But the figures differ in the SAARC region. The strains covered in PCV-10 contribute on an average 50% of the IPD (varying from 37% in Pakistan to 62% in India), whereas the strains covered in PCV-13 contribute on an average 55% of the IPD (varying from 42% in Bangladesh to 70% in India), in the SAARC region, as per the present systematic review.

Although antibiotics play a crucial role in the management of pneumococcal infections, data on antibiotic susceptibility is limited in the SAARC region [24], [34]. In the present systematic review, we found a 100% susceptibility rate to vancomycin, and 95% to levofloxaicn. The sensitivity to ceftriaxone was around 92.5%. Resistance to co-trimoxazole varied from 56–74% in different studies. Sri Lanka was the only SAARC country reporting a high non-susceptibility rate of almost 90% to penicillin, 60% to erythromycin, 50% to cefotaxime, and 26% to chloramphenicol (**Figure 3d**). We also studied the mean antibiotic susceptibility rates in different subgroups. The mean susceptibility rate was slightly less in children <5 years compared to ≤12 years (**Figure 3b**). When we compared the mean susceptibility rates during three different time periods (1990–2000, 2001–2010, and 2011–2013), a decreasing trend (increased non-susceptibility) was noted for co-trimoxazole, chloramphenicol, and erythromycin; cefotaxime and ceftriaxone showed no change; whereas an increasing trend was noted for penicillin (**Figure 3c**).

### Strengths & Limitations

The strength of present systematic review is the inclusion of a large number of studies spanning over more than 22 years period to estimate the average serotype distribution and pattern of antimicrobial resistance. Studies of short duration risk over- or underestimating serotype coverage due to inability to take into account the periodicity of serotypes [35]. Some of the limitations are common or inherent in systematic reviews in general, such as the potential for selection bias due to inclusion and exclusion criteria. In an effort to minimize the selection bias, we defined inclusion and exclusion criteria *a priori* to create a final data set aligned with our primary question of interest. We could not find any study from the three SAARC countries (Afghanistan, Maldives, and Bhutan), and only a single study was conducted each in Pakistan and Sri Lanka. Finally, some of the included studies were conducted almost a decade back during which the epidemiology of *S. pneumoniae* might have changed to a great extent.

## Conclusions

*Streptococcus pneumoniae* causes substantial disease burden in the children of SAARC countries with a wide variation in prevalent serotypes and antibiotic resistance patterns. Due to paucity of recent data outlining serotypes causing IPD and pattern of antimicrobial resistance in majority of the SAARC countries, urgent large size prospective studies are needed to formulate recommendations for specific pneumococcal vaccine introduction and usage of antimicrobial agents in these regions. PCV-10 may be suitable for countries like India, Nepal, Bangladesh, and Sri Lanka, whereas PCV-13 may be more suitable for Pakistan.

### Ethical Approval

An ethics statement was not required for this work.

## Supporting Information

Appendix S1 **Details of searches for pubmed and embase.**
(DOCX)

Checklist S1 **PRISMA Checklist.**
(DOC)
