# Supplementary material for: Distribution of Serotypes, Vaccine Coverage, and Antimicrobial Susceptibility Pattern of Streptococcus Pneumoniae in Children Living in SAARC Countries: A Systematic Review
Source: PLoS One. 2014 Sep 30;9(9):e108617. doi: 10.1371/journal.pone.0108617 (PMC4182530; doi:10.1371/journal.pone.0108617)
Supplement: Appendix S1 — Details of searches for pubmed and embase. (DOCX) [file pone.0108617.s001.docx]

**Appendix 1**

**PUBMED Search Strategy**

1. ("residence characteristics"[MeSH Terms] OR ("residence"[All Fields] AND "characteristics"[All Fields]) OR "residence characteristics"[All Fields] OR "community"[All Fields]) AND acquired[All Fields] AND ("pneumonia"[MeSH Terms] OR "pneumonia"[All Fields])
2. ("residence characteristics"[MeSH Terms] OR ("residence"[All Fields] AND "characteristics"[All Fields]) OR "residence characteristics"[All Fields] OR "community"[All Fields]) AND acquired[All Fields] AND ("meningitis"[MeSH Terms] OR "meningitis"[All Fields])
3. "meningitis"[MeSH Terms] OR "meningitis"[All Fields]
4. "pneumonia"[MeSH Terms] OR "pneumonia"[All Fields]
5. (#2) AND #3
6. (#1) AND #4
7. "child"[MeSH Terms] OR "child"[All Fields]
8. "child"[MeSH Terms] OR "child"[All Fields] OR "children"[All Fields]
9. (#7) AND #8
10. ((#6) OR #5) AND #9
11. (#6) OR ("streptococcus pneumoniae"[MeSH Terms] OR ("streptococcus"[All Fields] AND "pneumoniae"[All Fields]) OR "streptococcus pneumoniae"[All Fields])
12. (#6) AND ("streptococcus pneumoniae"[MeSH Terms] OR ("streptococcus"[All Fields] AND "pneumoniae"[All Fields]) OR "streptococcus pneumoniae"[All Fields])
13. (#5) AND ("streptococcus pneumoniae"[MeSH Terms] OR ("streptococcus"[All Fields] AND "pneumoniae"[All Fields]) OR "streptococcus pneumoniae"[All Fields])
14. (#12) AND #9
15. (#13) AND #9
16. (#14) AND "Sri Lanka"[All Fields]
17. (#15) AND "Sri Lanka"[All Fields]
18. (#14) AND "Pakistan"[All Fields]
19. (#15) AND "Pakistan"[All Fields
20. (#14) AND "Bangladesh"[All Fields]
21. (#15) AND "Bangladesh"[All Fields]
22. (#14)AND "Nepal"[All Fields]
23. (#15)AND "Nepal"[All Fields]
24. (#14)AND "Maldives"[All Fields]
25. (#15)AND "Maldives"[All Fields]
26. (#14)AND "Bhutan"[All Fields]
27. (#15)AND "Bhutan"[All Fields]
28. (#14)AND "India"[All Fields]
29. (#15)AND "India"[All Fields]
30. (#14)AND "Afghanistan"[All Fields]
31. (#15)AND "Afghanistan"[All Fields]
32. (#14) AND "South Asia"[All Fields]

**EMBASE Search Strategy**

26. #8 AND #25

25. 'South Asia'/exp OR 'South Asia'

24. #8 AND #23

23. 'afghanistan'/exp OR 'afghanistan'

22. #8 AND #21

21. 'bhutan'/exp OR 'bhutan'

20. #8 AND #19

19. 'bangladesh'/exp OR 'bangladesh'

18. #8 AND #17

17. 'nepal'/exp OR 'nepal'

16. #8 AND #15

15. 'maldives'/exp OR 'maldives'

14. #8 AND #13

13. 'sri lanka'/exp OR 'sri lanka'

12. #8 AND #11

11. 'pakistan'/exp OR 'pakistan'

10. #8 AND #9

9. 'india'/exp OR 'india'

8. #4 OR #6

7. #2 AND #6

6. 'invasive pneumococcal infections' AND ('meningitis'/exp OR meningitis)

5. 'invasive pneumococcal infections'

4. #2 AND #3

3. 'community acquired pneumonia'/exp OR 'community acquired pneumonia'

2. 'child'/exp OR child AND ('children'/exp OR children) OR 'infant'/exp OR infant OR pediatric

1. 'pneumonia'/exp OR pneumonia
